# Stent Retriever Thrombectomy for Anterior vs. Posterior Circulation Ischemic Stroke: Analysis of the STRATIS Registry

**DOI:** 10.3389/fneur.2021.706130

**Published:** 2021-08-23

**Authors:** Reza Jahan, David S. Liebeskind, Osama O. Zaidat, Nils H. Mueller-Kronast, Michael T. Froehler, Jeffrey L. Saver

**Affiliations:** ^1^Division of Interventional Neuroradiology, Department of Radiology, David Geffen School of Medicine, University of California, Los Angeles, Los Angeles, CA, United States; ^2^Department of Neurology, David Geffen School of Medicine, University of California, Los Angeles, Los Angeles, CA, United States; ^3^Department of Neurology, Mercy Health St. Vincent Hospital, Toledo, OH, United States; ^4^Advanced Neuroscience Network/Tenet South Florida, Boynton Beach, FL, United States; ^5^Cerebrovascular Program, Vanderbilt University Medical Center, Nashville, TN, United States

**Keywords:** ischemic stroke, mechanical thrombectomy, STRATIS registry, posterior circulation, anterior circulation

## Abstract

**Background and Purpose:** The benefits of mechanical thrombectomy (MT) in vertebrobasilar artery occlusions have not been well-studied. We compared clinical, procedural, and safety outcomes of MT for posterior circulation (PC) vs. anterior circulation (AC) occlusions among patients in the STRATIS registry.

**Methods:** Data from STRATIS including patient demographics, procedural characteristics, and outcomes including symptomatic intracranial hemorrhage (sICH) at 24 h, serious adverse events (SAE), substantial reperfusion [modified thrombolysis in cerebral infarction (mTICI) 2b/3], 90-day functional independence [modified Rankin Scale (mRS) 0–2], and 90-day mortality were analyzed. Univariate logistic regression was used to calculate predictors of good clinical outcome.

**Results:** Of 984 STRATIS patients, 43 (4.4%) patients with PC occlusions [mean age 63.0 ± 13.6, 25.6% (11/43) female] and 932 (94.7%) with AC occlusions [mean age 68.5 ± 14.8, 46.9% (437/932) female] were included for analysis. Median National Institutes of Health Stroke Scale (NIHSS) scores at baseline were 17.0 (13.0, 12.0) for the AC group and 12.0 (11.0, 24.0) for the PC group. Time from onset to procedure end was longer for the PC group [median (IQR): 322.0 min (255.0–421.0) vs. 271.0 min (207.0–360.0); *p* = 0.007]. PC and AC groups had similar rates of substantial reperfusion [89.2% (33/37) vs. 87.7% (684/780)], procedure-related SAE [0.0% (0/43) vs. 1.7% (16/932)], sICH [0.0% (0/38) vs. 1.5% (12/795)], 90-day functional independence [66.7% (26/39) vs. 55.9% (480/858)] and mortality [12.8% (5/39) vs. 15.8% (136/861)]. National Institutes of Health Stroke Scale score and patient sex were significant univariate predictors of good clinical outcome (*p* < 0.05).

**Conclusions:** Despite longer reperfusion times, MT in PC stroke has similar rates of 90-day functional independence with no significant difference in procedure-related SAE, sICH, or mortality, supporting the use of MT in PC acute ischemic stroke (AIS).

**Clinical Trial Registration:**https://www.clinicaltrials.gov, Identifier: NCT02239640.

## Introduction

Arterial vertebrobasilar artery occlusions constitute ~20% of cases of acute ischemic stroke (AIS) (1), with 1% due to basilar artery occlusion, and are associated with a high mortality rate (2, 3). Randomized trials of mechanical thrombectomy (MT) in patients with anterior circulation (AC) large vessel occlusion (LVO) have demonstrated superior clinical safety and efficacy compared to medical therapy (4–9). In contrast, for patients with vertebrobasilar occlusions, accumulating data remain non-definitive. Large registries have generally signaled better outcomes with MT but are subject to bias across therapeutic indications due to non-randomized treatment assignment (10–13). In addition, randomized trials of mechanical intervention for management of vertebrobasilar occlusions have remained inconclusive (14, 15). The purpose of this study was to report the technical outcomes of endovascular intervention along with safety and efficacy measures among patients with posterior circulation (PC) LVO ischemic stroke who were treated with stent retriever-based MT in the multicenter, nationwide, prospective, United States (US) STRATIS registry (Systematic Evaluation of Patients Treated With Neurothrombectomy Devices for Acute Ischemic Stroke) (16), and compare these results to those treated for AC LVO ischemic stroke.

## Materials and Methods

### Study Population

The primary results of the STRATIS registry have been reported (16). STRATIS was a prospective, multicenter, non-randomized, observational registry evaluating the use of Solitaire Revascularization Device (Medtronic, Dublin, Ireland) in patients presenting with AIS in the setting of intracranial LVO. Inclusion criteria were: (1) any confirmed intracranial LVO with associated ischemic symptoms; (2) planned stent retriever-based thrombectomy; (3) treatment within 8 h of stroke onset; (4) modified Rankin Scale (mRS) score ≤1 prior to stroke onset; and (5) pre-treatment National Institutes of Health Stroke Scale (NIHSS) score ≥8 and ≤30. Written informed consent was obtained from patients before enrollment.

### Patient Characteristics, Imaging Features, Technical, Efficacy, and Safety Outcomes

Patient characteristics analyzed included age, baseline NIHSS, baseline mRS score, and medical history including history of atrial fibrillation and/or stroke etiology. Imaging characteristics analyzed included baseline ASPECTS (Alberta Stroke Program Early CT Score, AC patients only), site of occlusion, and baseline image screening modality. Workflow metrics included measurement of the time between (1) onset to arrival at enrolling hospital, (2) onset to the administration of intravenous (IV) tissue plasminogen activator (tPA), (3) arrival to IV-tPA, (4) onset to arterial puncture, (5) arrival to arterial puncture, (6) imaging to arterial puncture, (7) IV-tPA to arterial puncture, (8) puncture to procedure end, and (9) onset to procedure end. Technical outcomes included reperfusion as measured on the modified thrombolysis in cerebral infarction (mTICI) score post-procedure. Substantial reperfusion was defined as mTICI 2b/3 (16). Efficacy outcomes included mRS at 90 days and safety outcomes were symptomatic intracerebral hemorrhage (sICH) and 90-day mortality. Serious adverse events (SAE) were investigated. An mRS 0–2 at 90 days was defined as good functional outcome, and an mRS 0–1 at 90 days was defined as excellent functional outcome. Early neurological improvement was defined as NIHSS reduction ≥8 points or reaching 0–1 at 24 h.

### Statistical Analysis

Statistical analyses were conducted using SAS v9.4 (SAS Institute, Cary, NC). Standard descriptive statistics including mean, standard deviation (SD), and median with interquartile range (IQR) were used for measurement of continuous variables and frequency distributions for measurement of categorical variables. For between-group comparisons, *t*-tests were used for continuous variables, χ^2^ tests were used for categorical variables, and a Cochran-Mantel-Haenszel test was used for analyzing mRS shift. Univariate logistic regressions were used to calculate odds ratios (OR) in the predictors of outcome analysis. Two-tailed *p*-values <0.05 were considered statistically significant.

### Ethical Approval

This study was approved by the Institutional Review Board at each participating center.

## Results

Among 984 patients included in the STRATIS intent-to-treat (ITT) analysis population, 45 (4.6%) had PC occlusions, 939 (95.4%) had AC occlusions. A total of 8 (0.8%) patients had missing occlusion locations, and one additional patient with both AC and PC occlusion was excluded, resulting in a total of 975 patients included in the analysis (Table 1). Of the 43 patients treated for PC stroke, 41 (95.3%) had basilar artery occlusions. Compared to the AC patient cohort, the PC cohort was younger (63.0 ± 13.6 vs. 68.5 ± 14.8 years; *p* = 0.017), less often female [25.6% (11/43) vs. 46.9% (437/932); *p* = 0.006], had fewer patients with atrial fibrillation/flutter [11.6% (5/43) vs. 38.8% (362/932); *p* < 0.001], and more patients with history of prior hemorrhagic stroke [4.7% (2/43) vs. 0.8% (7/932); *p* = 0.009]. There was a significant difference in stroke etiology (*p* = 0.006), where the PC group had more strokes due to large artery disease [38.1% (16/42) vs. 18.5% (160/863)] and the AC group had more cardioembolic strokes [49.1% (424/863) vs. 33.3% (14/42)].

**Table 1:** Baseline characteristics of patients treated in the anterior vs. posterior circulation.

**Characteristic**	**Anterior mean ± SD (*N*) median (IQR) or % (*n*/*N*)**	**Posterior mean ± SD (*N*) median (IQR) or % (*n*/*N*)**	***T*-test or chi-squared *P*-value**
**Age (years)**	68.5 ± 14.8 (932) 69.8 (60.0, 79.9)	63.0 ± 13.6 (43) 60.7 (54.4, 74.3)	**0.017**
**Female**	46.9% (437/932)	25.6% (11/43)	**0.006**
**Medical history**			
Atrial fibrillation/flutter	38.8% (362/932)	11.6% (5/43)	** <0.001**
Hypertension	73.0% (680/932)	62.8% (27/43)	0.144
Diabetes mellitus	25.4% (237/932)	30.2% (13/43)	0.481
Myocardial disease/CAD	27.6% (257/932)	23.3% (10/43)	0.535
Hyperlipidemia	42.5% (396/932)	37.2% (16/43)	0.493
Peripheral artery disease	3.9% (36/932)	2.3% (1/43)	0.606
Carotid artery disease	7.8% (73/932)	9.3% (4/43)	0.727
Current or previous tobacco use	52.9% (443/838)	46.2% (18/39)	0.412
**Neurological history**			
Prior ischemic stroke	12.7% (118/932)	14.0% (6/43)	0.804
Prior hemorrhagic stroke	0.8% (7/932)	4.7% (2/43)	**0.009**
Prior TIA	5.9% (55/932)	4.7% (2/43)	0.733
Brain aneurysm	1.1% (10/932)	0.0% (0/43)	0.495
**Pre-stroke mRS**			0.714
0	76.0% (708/932)	81.4% (35/43)	
1	21.2% (198/932)	16.3% (7/43)	
2	2.8% (26/932)	2.3% (1/43)	
**NIHSS at baseline**	17.3 ± 5.4 (932) 17.0 (13.0, 21.0)	16.3 ± 7.3 (43) 12.0 (11.0, 24.0)	0.371
**ASPECTS per imaging core lab**			
Overall	8.2 ± 1.6 (756) 8.0 (8.0, 9.0)	Not documented	
0–5	7.4% (56/756)	Not documented	
6–7	15.3% (116/756)	Not documented	
8–10	77.2% (584/756)	Not documented	
**Occlusion location**			** <0.001**
Basilar	0.0% (0/932)	95.3% (41/43)	
Carotid T	23.8% (222/932)	0.0% (0/43)	
MCA-M1	57.7% (538/932)	0.0% (0/43)	
MCA-M2	18.2% (170/932)	0.0% (0/43)	
MCA-M3	0.2% (2/932)	0.0% (0/43)	
PCA	0.0% (0/932)	2.3% (1/43)	
Vertebral	0.0% (0/932)	2.3% (1/43)	
**Etiology of stroke**			**0.006**
Cardioembolic	49.1% (424/863)	33.3% (14/42)	
Large Artery	18.5% (160/863)	38.1% (16/42)	
Unknown	32.3% (279/863)	28.6% (12/42)	

*Bold values represent statistically significant differences. SD, standard deviation; IQR, interquartile range; CAD, coronary artery disease; TIA, transient ischemic attack; mRS, modified Rankin Scale; NIHSS, National Institutes of Health Stroke Scale; ASPECTS, Alberta Stroke Program Early CT Score; ICA, internal carotid artery; MCA, middle cerebral artery; PCA, posterior cerebral artery*.

Procedure and workflow characteristics are presented in Table 2. Fewer patients in the PC group received IV-tPA [41.9% (18/43) vs. 64.9% (604/930); *p* = 0.002]. The PC patient cohort more frequently underwent general anesthesia [57.5% (23/40) vs. 30.9% (242/784); *p* < 0.001], and had fewer cases with adjunctive balloon-guided catheter use [20.9% (9/43) vs. 60.0% (559/932); *p* < 0.001]. The mean number of device passes was similar for both cohorts (1.9 ± 1.2). The PC group had a longer onset-to-arterial puncture time [median (IQR): 257.0 min (187.0–355.0) vs. 207.0 min (147.0–289.5); *p* = 0.018], longer puncture-to-procedure end [median (IQR): 66.5 min (47.0–99.0) vs. 57.0 min (39.0–82.0); *p* = 0.046], and longer onset-to-procedure end [median (IQR): 322.0 min (255.0–421.0) vs. 271.0 min (207.0–360.0); *p* = 0.007]. Safety and efficacy outcomes of the endovascular intervention are reported in Table 3. Substantial reperfusion (mTICI 2b/3) adjudicated by the imaging core lab was not significantly different between groups [PC, 89.2% (33/37) vs. AC, 87.7% (684/780); adjusted *p* = 0.915]. However, there was a difference in core lab adjudicated post-procedure mTICI values (adjusted *p* = 0.001), where the PC group had a higher proportion of patients with final mTICI 3 [40.5% (15/37) vs. 11.3% (88/780)].

**Table 2:** Procedure and workflow characteristics.

**Characteristic**	**Anterior mean ± SD (*N*) median (IQR) or % (*n*/*N*)**	**Posterior mean ± SD (*N*) median (IQR) or % (*n*/*N*)**	***T*-test or chi-squared *P*-value**
**IV-tPA delivered**	64.9% (604/930)	41.9% (18/43)	**0.002**
**Screening imaging modality**			**0.347**
CT	92.6% (800/864)	97.3% (36/37)	
MR	2.1% (18/864)	2.7% (1/37)	
MR and CT	5.3% (46/864)	0.0% (0/37)	
**General anesthesia**	30.9% (242/784)	57.5% (23/40)	** <0.001**
**Use of balloon guide catheter**	60.0% (559/932)	20.9% (9/43)	** <0.001**
**Use of adjuvant therapy**			**0.031**
Carotid angioplasty or stenting	13.1% (122/932)	0% (0/43)	
Intracranial angioplasty or stenting	2.5% (23/932)	4.7% (2/43)	
Neither	84.4% (787/932)	95.3% (41/43)	
**Number of device passes**	1.9 ± 1.2 (932) 1.0 (1.0, 2.0)	1.9 ± 1.2 (43) 2.0 (1.0, 2.0)	0.774
**Onset to arrival at enrolling hospital (min)**	148.7 ± 100.6 (858) 135.0 (57.0, 220.0)	179.3 ± 100.7 (35) 180.0 (86.0, 271.0)	0.078
**Onset to tPA administration (min)**	113.0 ± 50.5 (599) 100.0 (78.0, 139.0)	122.3 ± 42.5 (18) 116.0 (90.0, 139.0)	0.441
**Arrival to tPA administration (min)**	42.1 ± 26.6 (326) 37.5 (26.0, 52.0)	44.4 ± 24.6 (9) 38.0 (29.0, 46.0)	0.793
**Onset to arterial puncture (min)**	224.6 ± 99.6 (924) 207.0 (147.0, 289.5)	261.3 ± 98.5 (43) 257.0 (187.0, 355.0)	**0.018**
**Arrival to puncture (min)**	79.5 ± 48.5 (857) 72.0 (46.0, 101.0)	96.6 ± 62.7 (36) 94.0 (57.0, 118.0)	0.114
**Imaging to puncture (min)**	70.4 ± 44.8 (782) 62.0 (39.0, 91.0)	93.5 ± 68.1 (34) 82.0 (39.0, 118.0)	0.059
**tPA to puncture (min)**	60.4 ± 42.5 (345) 51.0 (31.0, 77.0)	84.6 ± 82.4 (11) 63.0 (36.0, 70.0)	0.354
**Puncture to procedure end (min)**	65.0 ± 36.8 (911) 57.0 (39.0, 82.0)	76.6 ± 36.2 (42) 66.5 (47.0, 99.0)	**0.046**
**Onset to procedure end (min)**	289.7 ± 107.0 (919) 271.0 (207.0, 360.0)	335.1 ± 100.4 (42) 322.0 (255.0, 421.0)	**0.007**

*Bold values represent statistically significant differences. CT, Computed tomography; IV-tPA, intravenous tissue plasminogen activator; MR, Magnetic resonance; SD, standard deviation; IQR, interquartile range*.

**Table 3:** Technical, clinical, and safety outcomes.

**Outcome**	**Anterior mean ± SD (*N*) median (IQR) or % (*n*/*N*)**	**Posterior mean ± SD (*N*) median (IQR) or % (*n*/*N*)**	***T*-test or chi-squared *P*-value**	Adjusted *P*-value^*^
**mTICI post-procedure (core lab)**			** <0.001**	**0.001**
0	2.7% (21/780)	2.7% (1/37)		
1	0.9% (7/780)	2.7% (1/37)		
2a	8.7% (68/780)	5.4% (2/37)		
2b	76.4% (596/780)	48.6% (18/37)		
3	11.3% (88/780)	40.5% (15/37)		
**Substantial reperfusion (mTICI 2b/3) (imaging core lab)**	87.7% (684/780)	89.2% (33/37)	0.786	0.915
**Embolization to new territory**	0.9% (7/780)	0.0% (0/37)	0.563	0.362
**Categorical mRS at 90 days**			0.515	0.484
0	20.6% (177/858)	28.2% (11/39)		
1	22.5% (193/858)	20.5% (8/39)		
2	12.8% (110/858)	17.9% (7/39)		
3	14.1% (121/858)	7.7% (3/39)		
4	10.0% (86/858)	5.1% (2/39)		
5	4.1% (35/858)	7.7% (3/39)		
6	15.9% (136/858)	12.8% (5/39)		
**Good functional outcome (mRS 0–2) at 90 days**	55.9% (480/858)	66.7% (26/39)	0.187	0.207
**Excellent outcome (mRS 0–1) at 90 days**	43.1% (370/858)	48.7% (19/39)	0.491	0.329
**Early neurological improvement: NIHSS reduction** **≥8 points or reaching 0–1 at 24 h**	54.7% (456/834)	56.8% (21/37)	0.804	0.524
**Mortality at 90 days**	15.8% (136/861)	12.8% (5/39)	0.617	0.547
**Device-related SAE**	0.2% (2/932)	0.0% (0/43)	0.761	0.271
**Index procedure-related SAE**	1.7% (16/932)	0.0% (0/43)	0.386	0.741
**sICH at 24 h**	1.5% (12/795)	0.0% (0/38)	0.446	0.797
**PH-2 at 24 h**	2.6% (21/795)	0.0% (0/38)	0.310	0.594

*Bold value denotes a statistically significant difference. SD, standard deviation; IQR, interquartile range; mTICI, modified Thrombolysis in Cerebral Infarction; mRS, modified Rankin Scale; NIHSS, National Institutes of Health Stroke Scale; SAE, severe adverse event; ICH, intracranial hemorrhage; PH, parenchymal hematoma, sICH, symptomatic intracerebral hemorrhage*.

* *P-values are adjusted for age, NIHSS at baseline, gender, time from onset to arrival, and tPA administration. Other outcome variables are shown with unadjusted p-values due to zero or one event in the posterior group making adjusted modeling infeasible*.

**Figure 1:**
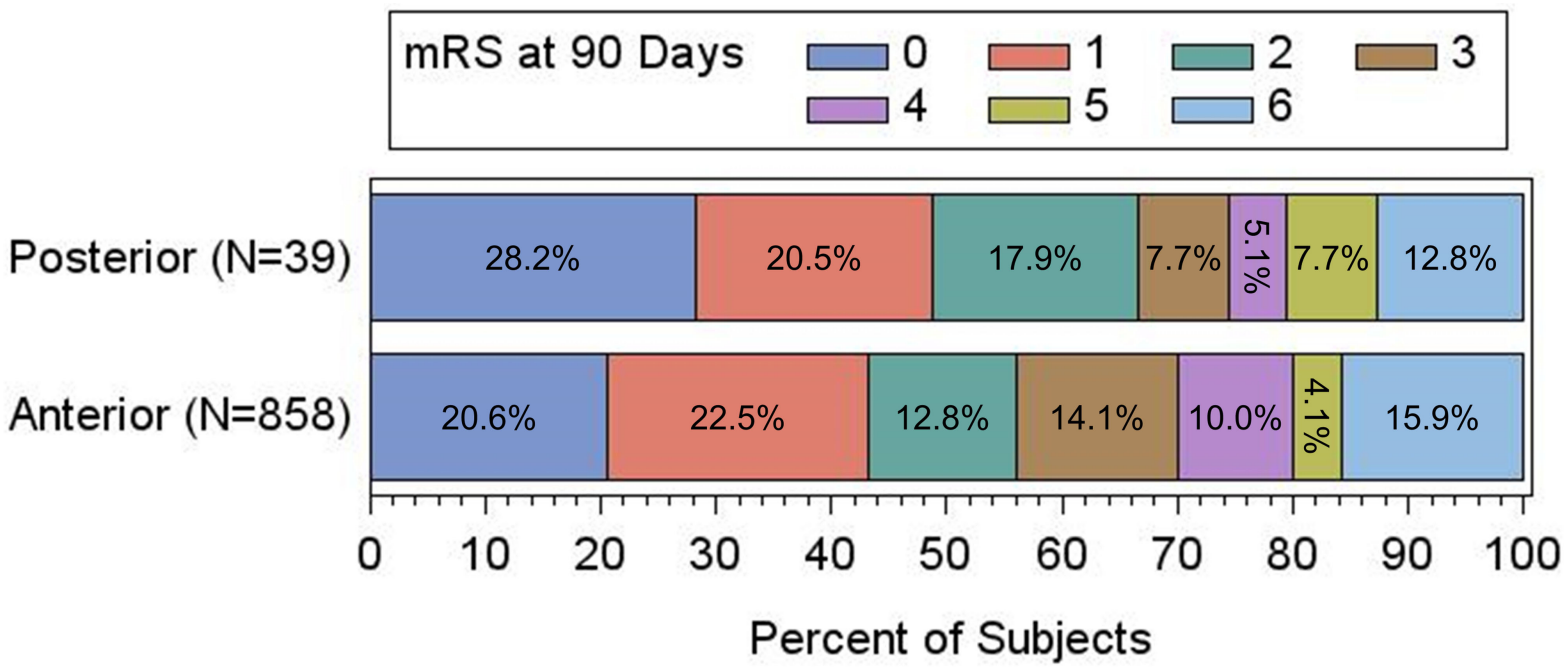
Adjusted clinical outcomes at 90 days based on mRS, presented as percentage of the total in anterior vs. posterior circulation patients. There is no significant difference between the two groups by shift analysis (*P* = 0.366 by Cochran-Mantel-Haenszel test).

There was no significant difference in 90-day mRS scores between the two populations (Table 3; Figure 1), with PC patients having nominally higher rates of good functional outcome (mRS 0–2) [66.7% (26/39) vs. 55.9% (480/858); adjusted *p* = 0.207] as well as excellent functional outcome (mRS 0–1) [48.7% (19/39) vs. 43.1% (370/858); adjusted *p* = 0.329]. There was no difference in the rate of sICH [PC, 0.0% (0/38) vs. AC, 1.5% (12/795); adjusted *p* = 0.797], procedure-related SAE [PC, 0.0% (0/43) vs. AC, 1.7% (16/932); adjusted *p* = 0.741], or 90-day mortality [PC, 12.8% (5/39) vs. AC, 15.8% (136/861); adjusted *p* = 0.547].

Among patients with PC stroke, rates of 90-day good functional outcome (mRS 0–2) with vs. without administration of IV-tPA were 82.4% (14/17) vs. 54.5% (12/22), *p* = 0.07 (Table 4). Further, amongst PC stroke patients, rates of a good outcome were similar in the onset-to-arterial puncture time windows of <3 h: 77.8% (7/9); 3–5 h 57.9% (11/19); >5 h 72.7% (8/11), *p* = 0.52. A total of 11 PC patient covariates were examined to ascertain if they may serve as a predictor for good clinical outcome (Table 5). Baseline NIHSS score was associated with mRS 0–2 at 90-days [OR 0.84, 95% confidence interval (CI): 0.75–0.94; *p* = 0.003], as was male sex [OR 4.71, 95% CI: 1.03–21.65; *p* = 0.046]. As only two patient characteristics were univariately associated, multivariate analysis was not undertaken.

**Table 4:** Proportion of patients with 90-day functional independence (mRS 0–2) based on procedure characteristics among the PC group.

**Characteristic**	**mRS 0–2% (*n*/*N*)**	**Chi-squared *P*-value**
**IV t-PA**		0.07
Delivered	82.4% (14/17)	
Not delivered	54.5% (12/22)	
**Onset-to-arterial puncture time**		0.52
<3 h	77.8% (7/9)	
3–5 h	57.9% (11/19)	
>5 h	72.7% (8/11)	

*IV-tPA, intravenous tissue plasminogen activator*.

**Table 5:** Predictors of good functional outcome (mRS 0–2) in patients with posterior circulation ischemic stroke (univariate analysis).

**Predictor**	**Odds ratio**	**LCL**	**UCL**	***p*-value**
**Age (per year)**	1.01	0.96	1.06	0.730
**NIHSS at baseline (per point)**	0.84	0.75	0.94	**0.003**
**Male (vs female)**	4.71	1.03	21.65	**0.046**
**Onset to arterial puncture (per minute)**	1.00	0.99	1.01	0.893
**IV-tPA administration (yes vs. no)**	3.89	0.87	17.48	0.077
**Systolic BP at baseline (per point)**	1.00	0.97	1.03	0.892
**Hypertension (yes vs. no)**	0.71	0.17	2.94	0.638
**Atrial fibrillation (yes vs. no)**	2.18	0.22	21.79	0.506
**Diabetes mellitus (yes vs. no)**	1.76	0.39	8.08	0.465
**Hyperlipidemia (yes vs. no)**	1.65	0.40	6.77	0.487
**mTICI 2b/3 post-procedure (yes vs. no)**	0.50	0.05	5.39	0.568

*Bold values represent statistically significant differences. LCL, lower confidence level; UCL, upper confidence level; NIHSS, National Institutes of Health Stroke Scale; vs., versus; IV-tPA, intravenous tissue plasminogen activator; BP, blood pressure; mTICI, modified Thrombolysis in Cerebral Infarction*.

## Discussion

This analysis of the prospective STRATIS registry showed stent retriever MT for LVO in the PC yielded similar procedural and clinical outcomes in comparison to the AC, with high rates of substantial reperfusion, and good functional outcome at 3 months, and low rates of sICH, procedure-related SAE, and mortality, suggesting the safety and efficacy of MT in patients with PC AIS. Furthermore, the rate of final complete reperfusion was significantly higher in PC vs. AC AIS patients with LVO.

Two recently published meta-analyses examined outcomes associated with MT for PC occlusion (17, 18), demonstrating lower functional independence at 90 days and a higher mortality risk among patients with PC strokes, but comparable recanalization rates and lower rates of intracranial hemorrhage and sICH. The similar rate of 90-day functional outcome in PC vs. AC patients observed in this study may be explained by the relatively younger patient age. It should also be noted that, although baseline NIHSS scores in PC and AC patients were comparable, it is widely recognized that the NIHSS may underestimate deficit severity in PC strokes as its component items are heavily weighted toward deficits common in AC strokes such as aphasia and hemiparesis. On the other hand, signs of PC stroke, including bulbar deficits and ataxia, receive fewer points on this scale (19); therefore, impairments at baseline may be worse in PC patients than indicated by the NIHSS.

The observation that PC patients received IV-tPA less frequently in comparison to AC patients may be explained by several factors, including delay in diagnosing PC occlusions as they have a wide range of clinical presentations, often with bilateral or uncommon cerebrovascular symptoms—which may make definitive diagnosis of PC stroke difficult (20). Similar factors are likely drivers of delays to treatment times observed in PC compared to AC patients. Furthermore, patients with PC stroke were more frequently intubated, which likely also contributed to delays in endovascular treatment start. The longer procedural times in PC patients may reflect the greater technical challenge in performing thrombectomy in occlusions that are more often admixtures of atherosclerosis and thrombus compared to thrombus alone.

No significant difference in functional outcome was observed for PC stroke patients treated in different onset-to-groin puncture time windows. For AC stroke, time to treatment initiation is an important factor affecting the outcome, since the fate of ischemic brain tissue depends on the duration of ischemic exposure (21). However, in AC stroke, ischemic core extent selection of patients attenuates the time-benefit relationship (8, 9, 21). Similarly, patient selection appears to attenuate time and outcome relations for PC stroke. In the Basilar Artery International Cooperation Study (BASICS) registry, investigators found that the prognosis was related to prolonged time from symptom onset, and patients with severe stroke at presentation treated beyond 9 h after onset had poor clinical outcome (22). However, patients with extensive infarct signs present at baseline were not excluded in the BASICS registry (10). In the Helsinki series of basilar artery occlusion (23), onset to treatment time (OTT), when adjusted for the extent of baseline ischemia, was not associated with poor outcome, and patients treated in the longest OTT interval had outcomes similar to those of patients treated earlier (24). The attenuated relation of time to treatment and outcome in PC stroke may also in part reflect the presence of a higher proportion of white matter in the brainstem, as the white matter is more resistant to ischemia (12). Furthermore, collateral flow through the posterior communicating or the cerebellar arteries may lead to slower evolution of irreversible ischemia further slowing stroke progression in PC stroke (19).

Surprisingly, there was no significant difference in mortality between the PC and AC groups and the rates of device- and procedure-related SAEs were similar. The mortality rate among the PC cohort in this study is excellent, especially when compared to other studies that have reported higher mortality rates among patients with basilar artery strokes. A recent study of data collected from patients with basilar artery occlusions in the prospective Basilar Artery International Cooperation Study (BASICS) reported a mortality rate of 38.3% (59/154) (15). Another recent retrospective study of prospectively collected data from patients with basilar artery occlusions by Ravindren et al. (25) reported a mortality rate of 36.8% (85/231) (25). The enrollment period for both studies includes earlier timeframes compared to STRATIS (BASICS = 2011–2019; Ravindren et al. = 2008–2019), which may have given STRATIS the advantage of more robust procedural optimization and treatment decision algorithms. Median time from onset to recanalization in Ravindren et al. was longer than the PC cohort in STRATIS (6.4 vs. 5.4 h), which is also known to impact clinical outcome (26). Median baseline NIHSS scores were also lower for the STRATIS PC cohort compared to BASICS (median baseline NIHSS = 21 in the endovascular therapy arm) and Ravindren et al. (median baseline NIHSS = 14). Finally, baseline factors outside of the data collected in STRATIS, such as collateral scores, are also known to impact clinical outcome in PC stroke patients (27). All of these factors may have contributed to the relatively low mortality rates we observed.

Despite the longer time to treatment in PC patients, our study showed nominally lower rates of sICH in PC patients, a finding consistent with prior studies assessing MT treatment (11, 12, 17, 18), as well as IV thrombolysis (20, 28). The lower rate of sICH likely reflects the small volumes of ischemia in PC vs. AC stroke, resulting in less pretreatment permeability abnormality of the blood-brain barrier (29).

The high reperfusion rate in the current study is comparable or better than in other studies of MT in PC stroke (11–14, 30). Unlike some studies that found a lower rate of reperfusion in the PC compared to the AC, in our cohort, there was no significant difference in substantial reperfusion rates between the two groups (30).

The initial NIHSS and male sex were predictors of outcome in our population of PC patients who underwent MT. While the modest cohort size limited the power to detect effects of other baseline patient features, the finding with regard to initial NIHSS does indicate a powerful relationship between baseline deficit severity and post-intervention long-term outcome. In addition to our findings, other authors have reported age, hypertension, diabetes mellitus, previous stroke, initial pc-ASPECTS, thalamic infarction, intracranial stenting, and treatment with glycoprotein IIb/IIIa inhibitors as outcome predictors in PC stroke thrombectomy (11, 13, 31–35).

There are few randomized trials of MT in the PC stroke and current evidence of benefit in this population is controversial. The BASICS registry (10), a prospective, observational, international registry of consecutive patients with acute symptomatic vertebrobasilar occlusions, suggested no definite superiority of intra-arterial thrombectomy over IV thrombolysis in patients with a mild-to-moderate deficit and, interestingly, reported a higher risk for poor outcomes when treated with MT (risk ratio: 1.49, 95% CI: 1.00–2.23). In patients with a severe deficit, outcomes were similar when treated with either MT or IV-tPA (risk ratio: 1.06, 95% CI: 0.91–1.22). It should be noted that the study did not specify a particular inclusion protocol, and the reasons for clinicians to select a specific treatment option are not clear and there may have been a bias toward more aggressive treatment in patients who were thought to have a worse prognosis, potentially influencing the outcome in the endovascular group. Finally, crossover to another treatment group because of clinical worsening or the absence of treatment response was not considered in the interpretation of the results. More recently, the final results of the BASICS study were reported (15). The study was designed as a multicenter, prospective, randomized, open-label treatment with blinded outcome assessment. Patients were assigned to intervention of medical management in a 1:1 ratio, stratified according to randomizing center, use of IV thrombolysis, and NIHSS score (<20 vs. ≥20). The study did not find a significant difference in clinical outcome, although endovascular therapy tended to be more effective in patients over age 70. Also, there was a significant difference in outcome favoring embolectomy in patients with moderate to severe stroke (NIHSS ≥10). Our study showing similar rates of reperfusion and good outcomes in PC vs. AC patients with no significant difference in safety events of sICH and mortality suggests a potential benefit in thrombectomy in this population of patients.

## Limitations

The major limitation of our study is its observational single-arm nature without a control group to compare the effectiveness of MT in terms of outcomes. As such, data regarding efficacy for the PC group is only in relation with AC strokes treated with MT. In addition, the number of patients in the PC group is relatively low, as STRATIS enrollment occurred between 2014 and 2016 and was intended to be on-label. During this period of time, surgeons were focused on treating patients with AC strokes, and PC strokes were considered off-label in most situations. During STRATIS, there were other clinical trials enrolling patients with PC strokes and most patients with this condition were diverted to those studies. Furthermore, basilar artery occlusions are rare, and the proportion of PC strokes in the population are in line with real-world data. We acknowledge that the small cohort of PC patients limits the robustness and generalizability of the evidence, and that larger studies are required to increase the PC stroke population. Additionally, there are baseline differences between the PC and AC groups, mainly due to stroke etiology, use of IV-tPA, and longer times to treatment in the PC group, the latter of which we attribute to the inherent challenge of treating PC strokes. Data related to rescue therapy and antithrombotic treatments were not collected in STRATIS.

Data quality was incomplete for some important predictors of outcome, such as the exact location of occlusions within the basilar artery (proximal, middle, distal portion) and vertebral arteries, collateral quality, and pc-ASPECTS scores (33, 36). The modest sample size limited study power to prognostic factors associated with favorable outcome after MT treatment. A larger sample of patients with PC strokes collected prospectively either in a randomized fashion or registry format is warranted to add depth to the existing literature on this topic.

## Conclusions

MT for PC AIS showed similar rates of reperfusion, favorable functional outcome, and safety endpoints in comparison with AC stroke. Longer times to treatment were noted in PC stroke patients, but did not adversely affect safety and clinical outcomes. These results provide support for the use of MT in AIS patients with posterior circulation occlusions, but definitive randomized clinical trial data are still needed to establish these observations.

## Data Availability Statement

The original contributions presented in the study are included in the article/supplementary material, further inquiries can be directed to the corresponding author.

## Ethics Statement

The studies involving human participants were reviewed and approved by the Institutional Review Board at each participating center. The patients/participants provided their written informed consent to participate in this study.

## Author Contributions

All authors listed have made a substantial, direct and intellectual contribution to the work, and approved it for publication.

## Conflict of Interest

RJ receives funding for his services as a scientific consultant regarding trial design and conduct to Medtronic/Covidien. DL serves as an imaging core lab consultant for Cerenovus, Genentech, Medtronic, and Stryker. OZ serves as a consultant for Neuravi/Cerenovus, Stryker, Penumbra, and Medtronic. NM-K serves as a scientific consultant regarding trial design and conduct to Medtronic. MF serves as a scientific consultant to Medtronic, Corindus, Balt, Cerenovus, Viz.ai, and Genentech, and has received research funding from the National Institutes of Health NIH, Stryker, Medtronic, Microvention, and Endophys. JS reports contracted hourly payments for service on clinical trial steering committee advising on rigorous trial design and conduct for Abbott, Medtronic, Stryker, and Cerenovus, and contracted stock options for service on clinical trial steering committee advising on rigorous trial design and conduct for Rapid Medical. The handling editor DH declared a past co-authorship/collaboration as part of large working groups with one of the authors OZ.

## Publisher's Note

All claims expressed in this article are solely those of the authors and do not necessarily represent those of their affiliated organizations, or those of the publisher, the editors and the reviewers. Any product that may be evaluated in this article, or claim that may be made by its manufacturer, is not guaranteed or endorsed by the publisher.

## Abbreviations

AC: anterior circulation
AIS: acute ischemic stroke
ASPECTS: Alberta Stroke Program Early CT Score
BASICS: Basilar Artery International Cooperation Study
BP: blood pressure
CAD: coronary artery disease
CT: computed tomography
ICA: internal carotid artery
ICH: intracranial hemorrhage
IQR: interquartile range
ITT: intent-to-treat
IV: intravenous
LCL: lower confidence level
LVO: large vessel occlusion
MCA: middle cerebral artery
MR: magnetic resonance
mRS: modified Rankin Scale
MT: mechanical thrombectomy
mTICI: modified thrombolysis in cerebral infarction
NIHSS: National Institutes of Health Stroke Scale
OR: odds ratios
PC: posterior circulation
PCA: posterior cerebral artery
PH: parenchymal hematoma
SAE: serious adverse events
SD: standard deviation
sICH: symptomatic intracranial hemorrhage
STRATIS: Systematic Evaluation of Patients Treated With Neurothrombectomy Devices for Acute Ischemic Stroke
TIA: transient ischemic attack
tPA: tissue plasminogen activator
UCL: upper confidence level.
